# Postinfektiöse reaktive Arthritis nach Chlamydieninfektion im Leistungssport: Klinisches Management und aktuelle Literaturübersicht

**DOI:** 10.1007/s00132-020-03935-3

**Published:** 2020-06-24

**Authors:** Sebastian Klingebiel, Horst Rieger, Georg Gosheger, Jan Christoph Theil, Carolin Rickert, Kristian Nikolaus Schneider

**Affiliations:** 1 Klinik für Allgemeine Orthopädie und Tumororthopädie, Universitätsklinikum Münster, Albert-Schweitzer Campus 1, 48149 Münster, Deutschland; 2 Klinik für Unfallchirurgie, Orthopädie, Handchirurgie und Sportmedizin, Clemenshospital Münster, Münster, Deutschland

**Keywords:** Athleten, Chlamydien, Gelenkerguss, SARA, Synovialitis, Athletes, Chlamydia, Joint-swelling, SARA, Synovialitis

## Abstract

**Hintergrund:**

Die reaktive Arthritis infolge einer Chlamydieninfektion ist eine seltene, aber wichtige Differenzialdiagnose der atraumatischen Gelenkschwellung. Eine verzögerte Diagnosestellung führt nicht selten zu langen Ausfallzeiten der sportlichen Aktivität, die gerade beim Leistungssportler weitreichende Konsequenzen haben kann.

**Ziel der Arbeit:**

Darstellung des klinischen Managements der postinfektiösen reaktiven Arthritis zur schnellen Diagnosestellung und zielgerichteten Behandlung bei symptomatischem Krankheitsverlauf.

**Material und Methoden:**

Literaturrecherche zu den Themen „Chlamydien“, „reaktive Arthritis“, „postinfektiöse Arthritis“ und „sexually acquired reactive arthritis“ einschließlich Darstellung von zwei klinischen Fällen der postinfektiösen reaktiven Arthritis nach Chlamydieninfektion aus dem Leistungssport.

**Ergebnisse und Fazit:**

Die reaktive Arthritis nach Chlamydieninfektion bei Leistungssportlern ist eine seltene Entität. Sie kann jedoch durchaus mit weitreichenden individuellen Folgen, insbesondere hinsichtlich möglicher Ausfallzeiten, im Sport einhergehen. Auch langfristige Folgen, wie chronische Gelenkschäden bei unterhaltener Synovialitis, müssen bedacht werden. Zur Diagnosestellung ist eine gezielte Anamneseerhebung und der direkte Erregernachweis im Gelenkpunktat durch die Polymerasekettenreaktion essenziell. Dies erlaubt eine sichere Diagnosestellung mit verzögerungsfreier Therapieeinleitung. Jedoch sind auch bei frühzeitigem Therapiebeginn protrahierte Krankheitsverläufe nicht ausgeschlossen.

## Abstract (en)

**Background:**

Reactive arthritis following infection with chlamydia is a rare but important differential diagnosis in atraumatic joint swelling. A delayed diagnosis often leads to prolonged periods of absence from physical activity. This can have serious consequences, especially for the career of competitive athletes.

**Objectives:**

Recommendation for the clinical management of postinfectious reactive arthritis for rapid diagnosis and targeted treatment in the symptomatic clinical course.

**Materials and methods:**

Review of the literature on the topics “chlamydia”, “reactive arthritis”, “postinfectious arthritis” and “sexually acquired reactive arthritis”, including presentation of two clinical cases of postinfectious reactive arthritis after chlamydia infection from competitive sports.

**Results and conclusion:**

Reactive arthritis following chlamydia infection in competitive athletes is a rare entity. However, it can be accompanied by far-reaching individual consequences, especially with regard to possible downtime in sports. Long-term consequences such as chronic joint damage in maintained synovitis must also be considered. In order to make a diagnosis, a specific anamnesis and the direct detection of the pathogen in the specimen of synovial fluid by polymerase chain reaction is essential. This allows a reliable diagnosis to be made with immediate initiation of therapy. However, a prolonged course of the disease cannot be excluded even if therapy is started in due time.

„A youth does not suffer from gout until after sexual intercourse“, so erkannte schon Hippokrates die potenziell pikante Genese der postinfektiösen reaktiven Arthritis [44]. Die oftmals mangelnde Berücksichtigung der Differenzialdiagnose einer SARA bei atraumatischer Gelenkschwellung führt bei dieser Entität nicht selten zu einer verzögerten Diagnosestellung oder Fehldiagnosen. Wir präsentieren zwei klinische Fälle von Leistungssportlern mit SARA des Kniegelenkes als Komplikation infolge einer Chlamydieninfektion und geben Empfehlungen zum klinischen Management dieses Erkrankungsbildes.

## Fall 1

Ein 21-jähriger Fußballspieler der Regionalliga stellte sich aufgrund einer atraumatischen und schmerzfreien Schwellung des rechten Kniegelenkes in unserer Klinik vor. Eine Alltagsbelastung war möglich. Sportliche Aktivitäten konnten jedoch aufgrund einer Bewegungseinschränkung des Kniegelenkes mit Spannungsgefühl nicht durchgeführt werden. Anamnestisch gab der Patient auf explizite Nachfrage an, 6 Wochen zuvor wegen einer urogenitalen Infektion antibiotisch behandelt worden zu sein. Ein positiver PCR-Nachweis von Chlamydia trachomatis im Urethralabstrich konnte vom Hausarzt angefordert werden.

In der klinischen Untersuchung präsentierte sich ein athletischer junger Mann mit ausgeprägtem Erguss des rechten Kniegelenkes. Es bestand ein leichtes Streckdefizit und endgradiges Flexionsdefizit von 20°. Des Weiteren zeigten sich in der klinischen Untersuchung stabile Bandverhältnisse und unauffällige Provokationstests, sowie fehlende lokale und systemische Infektstigmata.

**Figure 1:**
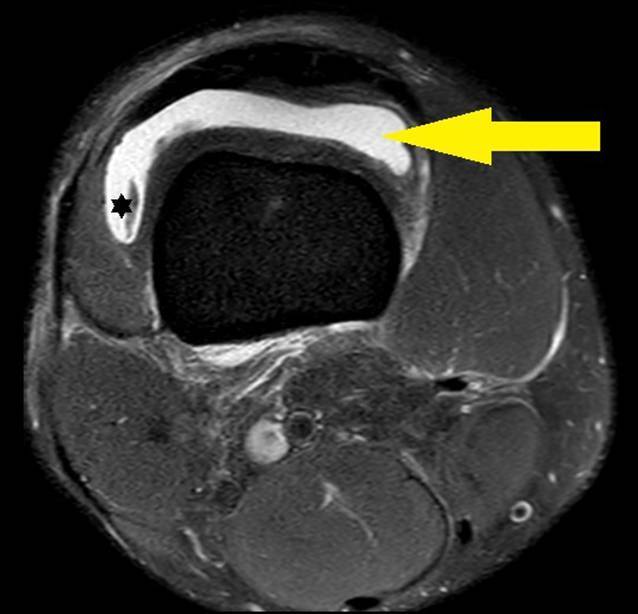

Die ergänzende MRT-Bildgebung (Abb. 1) zeigte einen ausgeprägten intraartikulären Erguss mit deutlicher Synovialitis. Weitere intraartikuläre Pathologien oder Verletzungen wesentlicher Strukturen konnten nicht nachgewiesen werden. In Zusammenschau der Befunde wurde die Diagnose einer postinfektiösen reaktiven Arthritis gestellt.

Aufgrund der auswärtig initiierten leitliniengerechten antibiotischen Therapie der Urethritis mittels Doxycyclin erfolgte nur eine symptomatische Behandlung der Monarthritis. Neben einer einwöchigen oralen Therapie mit NSAR (Etoricoxib 90 mg 1 × tgl.) wurden physikalischen Maßnahmen (Kryotherapie, manuelle Lymphdrainage) und Physiotherapie (propriozeptive Beübung, Remobilisation, muskuläre Kräftigung) durchgeführt. Hierunter kam es zu einer raschen Regredienz des Gelenkergusses. Die volle Sportfähigkeit war 6 Wochen nach Therapiebeginn der Gelenkbeschwerden gegeben. Sekundärkomplikationen blieben im weiteren Beobachtungszeitraum von 3 Jahren aus.

## Fall 2

Ein 25-jähriger Fußballspieler der Oberliga stellte sich aufgrund einer ausgeprägten, aber wenig schmerzhaften Schwellung des rechten Kniegelenkes in unserer Ambulanz vor. Am Vortag sei es im Rahmen eines Meisterschaftsspiels in einem Sprintduell zu kurzzeitig einschießenden Schmerzen des Kniegelenkes mit temporärem Instabilitätsgefühl gekommen. Die Belastung sei jedoch bis Spielende fortgeführt worden. Eine Kniedistorsion oder Fremdeinwirkung wurden verneint. Fieber bzw. B‑Symptomatik lagen nicht vor.

**Figure 2:**
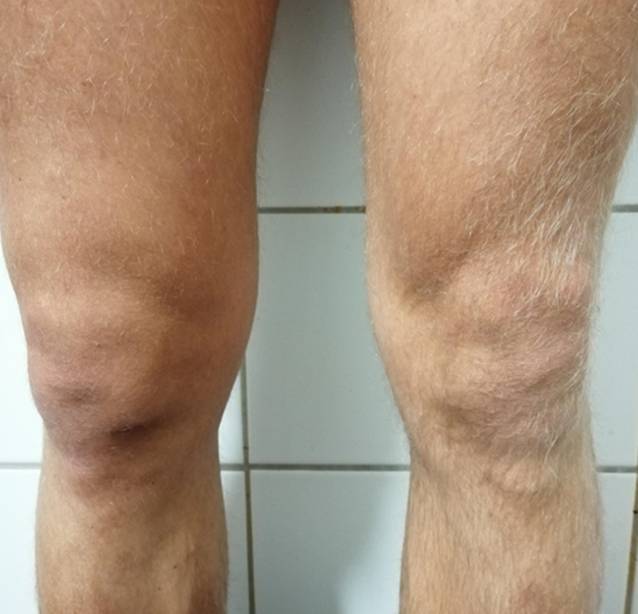

In der klinischen Untersuchung präsentierte sich ein sportlicher junger Mann in gutem Allgemeinzustand mit verstrichenen Konturen des rechten Kniegelenkes (Abb. 2). Es bestand ein leichtes Schonhinken mit Vermeidung der vollen Extension des betroffenen Gelenkes. Das Bewegungsausmaß betrug für die Extension/Flexion 0/10/110°. Neben dem ausgeprägtem Gelenkerguss ohne lokale Infektstigmata bestand ein leichter Hyperextensionsschmerz. Es lagen stabile Kollateral- und Kreuzbandverhältnisse vor. Sonographisch stellte sich ein intraartikulärer Erguss dar. Laborchemisch bestanden unauffällige Infektparamter (CRP, Leukozyten, BSG). Die ergänzende Borrelienserologie verblieb negativ.

**Figure 3:**
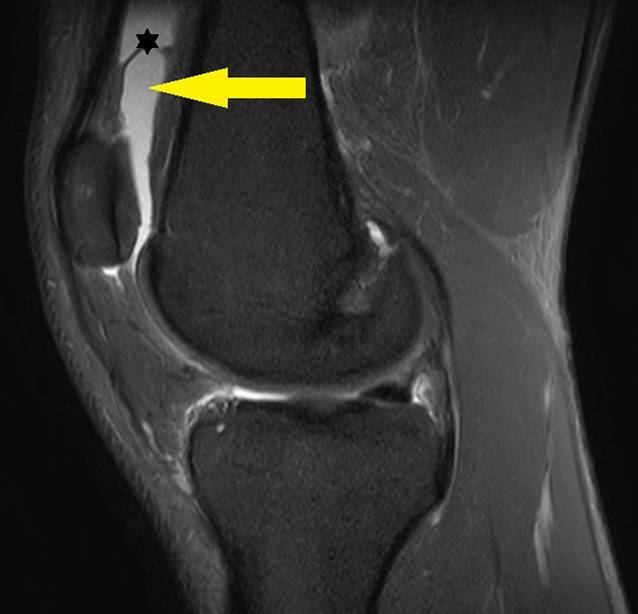

Wir initiierten eine orale antiphlogistische Therapie (Etoricoxib 90 mg 1 × tgl. für 7 Tage) und eine MRT-Bildgebung des rechten Kniegelenkes (Abb. 3). Letztere bestätigte einen ausgeprägten Gelenkerguss mit ausgeprägter Synovialitis bei ansonsten intakten Kniebinnenstrukturen. Es erfolgte zunächst eine therapeutische Punktion des Gelenkes ohne mikrobiologische Aufarbeitung bei Verdacht auf einen Reizerguss und nicht vorhandener Infektkonstellation.

**Figure 4:**
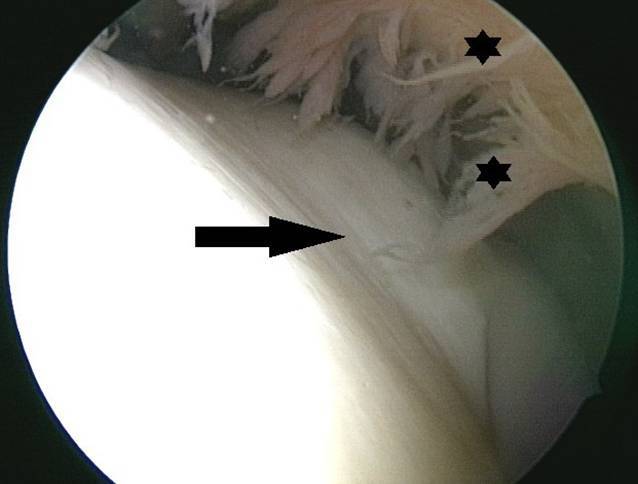

Nach rund 10 Tagen beklagte der Patient trotz initialer postpunktioneller Beschwerdebesserung eine identische Symptomatik im Rahmen eines erneut aufgetretenen Gelenkerguss. Es bestand seitens des Patienten der dringliche Wunsch einer weiteren operativen Abklärung, um eine schnelle Sportrückkehr nicht zu gefährden. Daher wurde eine diagnostische Arthroskopie angestrebt. Intraoperativ bestand bei intakten Kniebinnenstrukturen eine ausgeprägte Synovialitis (Abb. 4 und 5), sodass eine partielle Synovialektomie durchgeführt wurde. Histopathologisch wurde eine chronische Synovialitis mit ausgeprägter Inflammation bestätigt. Die konventionelle kulturelle mikrobiologische Untersuchung intraoperativer Proben der Synovia fiel negativ aus.

**Figure 5:**
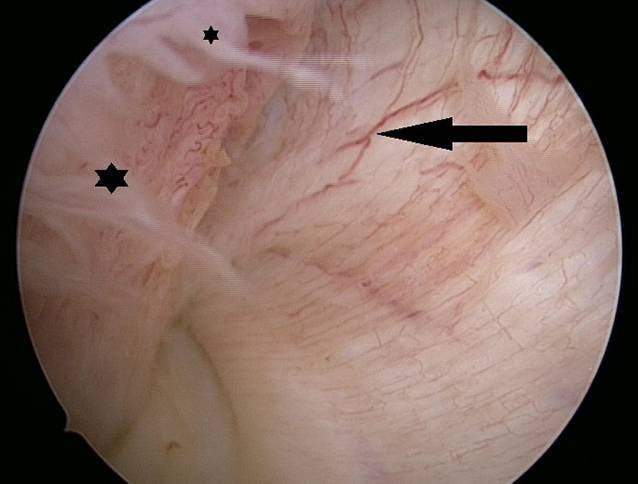

Aufgrund des protrahierten und therapierefraktären Verlaufes erfolgten erneute differentialdiagnostische Überlegungen. In diesem Rahmen schilderte der Patient auf mehrfache konkrete Nachfrage wechselnde Partnerschaften. Er habe seit etwa 2 Wochen vor Beginn der Knieschwellung gelegentlich urethralen Ausfluss gehabt, diesen Zustand jedoch als vorübergehend erachtet.

Ein kultureller Erregernachweis im Urethralabstrich und im Erststrahlurin gelang nicht. In der PCR des Urethralabstriches wurde jedoch *C. trachomatis* nachgewiesen. Serologisch waren die IgM- und IgG-Antikörper für *C. trachomatis* erhöht. Somit wurde die Diagnose einer sexuell erworbenen reaktiven Arthritis (SARA) gestellt. Wir initiierten die orale antibiotische Behandlung mit Doxycyclin. Die symptomatische und physiotherapeutische Behandlung der Monarthritis wurde fortgeführt.

Die urogenitale Symptomatik sistierte nach wenigen Tagen. Die Gelenkbeschwerden waren bis dato bereits regredient. Die Aufnahme der sportlichen Tätigkeit verlief jedoch noch über weitere Wochen frustran und konnte uneingeschränkt erst ca. 14 Wochen nach Diagnosestellung erfolgen. Diese zeitliche Latenz war insbesondere mit einem großen psychischen Leidensdruck verbunden.

## Material und Methoden

Unter Berücksichtigung der beiden klinischen Fälle erfolgte die Literaturrecherche zu den Themen „Chlamydien“, „reaktive Arthritis“, „postinfektiöse Arthritis“ und „sexually acquired reactive arthritis“ einschließlich Darstellung von zwei klinischen Fällen der postinfektiösen reaktiven Arthritis nach Chlamydieninfektion aus dem Leistungssport. Zudem erfolgte die Literaturrecherche hinsichtlich Daten von Chlamydieninfektionen und der postinfektiösen reaktiven Arthritis im Leistungssport.

## (Sexuell erworbene) reaktive Arthritis und HLA-B27-Assoziation

Die ReA wird der Familie der Spondylarthropathien zugeordnet [14]. Es handelt sich um eine akute oder chronische Synovialitis häufig großer und mittelgroßer Gelenke als Folge einer bakteriellen Gastroenteritis, nach Infektion der Atemwege oder des Urogenitaltraktes. Jedoch auch kleinere Gelenke können betroffen sein. Sofern die ReA sexuell erworben ist, liegt eine SARA (engl. „sexually acquired reactive arthritis“) vor [14]. Typischerweise setzen die Symptome 1–6 Wochen nach der Indexinfektion ein [37]. Bei Infektionen mit *C. trachomatis* infizieren Erregerbestandteile als „Agens“ die Synoviozyten und können lokal eine Inflammation verursachen [36]. Das Risiko, eine postinfektiöse ReA zu bekommen, liegt bei HLA-B27-positiven Patienten bis zu 50fach erhöht [29]. Die exakte Ursache hierfür ist noch unklar. Möglicherweise präsentieren HLA-B27-Träger ein Peptid aus einem Chlamydienprotein, das zu einer auf das körpereigene Gewebe kreuzreaktiven T‑Zellantwort führt, da die Sequenz des Chlamydienpeptids einem körpereigenen Peptid ähnelt (molekulare Mimikry). Alternativ könnten diese Kreuzreaktionen auch zu einer erhöhten Erregertoleranz führen [9], sodass die Verhinderung der Apoptose infizierter Zellen eine Infektpersistenz unterhalten kann [31].

## Mikrobiologische Aspekte von Chlamydien

Chlamydien sind obligat intrazelluläre Bakterien mit reduziertem Genom von etwa 1000 Genen [8]. Es sind verschiedene humanpathogene Chlamydienarten bekannt. *C. trachomatis* ist einer der bekanntesten Vertreter. Von *C. trachomatis* existieren mehrere Serovare. Die Serovare A–C verursachen das charakteristische Trachom, welches als schwere Keratokonjunktivitis bis zur Erblindung führen kann [5]. Die Serovare D–K induzieren neben den primären Urogenitalinfektionen sekundär potenzielle reaktive Entzündungsreaktionen der Synovia (SARA). Dies manifestiert sich klinisch als Mon- oder Oligoarthritis [5].

Chlamydien sind für ihren zweiphasigen Replikationszyklus auf eukaryonte Wirtszellen angewiesen. Zunächst werden infektiöse Elementarkörperchen von mukösen Zellen (z. B. Urothel) aufgenommen. Die Erreger können die Fusion von Phagosomen und Lysosomen verhindern, sodass die Bakterien sich der Immunantwort entziehen und intrazellulär überleben [12]. Die metabolisch wenig aktiven Elementarkörperchen transformieren zu stoffwechselaktiven Retikularkörperchen, um sich zu teilen. Die langfristig in menschlichen Zellen überlebenden Formen sind die unter Interferoneinfluss der humoralen Immunantwort entstehenden aberrierenden Retikularkörperchen [19]. Diese weisen einen sehr langsamen Vermehrungszyklus auf und können so eine chronische Inflammation unterhalten.

## Epidemiologie

Vorwiegend von einer SARA betroffen sind sexuell aktive junge Erwachsene zwischen dem 20. und 40. Lebensjahr. Männer weisen eine bis zu 3fach erhöhte Inzidenz auf [10]. Zu Beginn der 1970er-Jahre lag die Inzidenz für inflammatorische Gelenkerkrankungen bei 218/100.000, hiervon jeweils 6 % als reaktive Arthritis und Reiter-Trias (Uveitis, Urethritis, Arthritis) [20]. 2016 fassten Denison et al. die Prävalenz der SARA in einem systematischen Review mit 3,0–8,1 % zusammen [10]. Daten aus Finnland zeigten eine Prävalenz der Bevölkerung von 5,4/100.000 [32]. Bei etwa 1 % der Patienten mit Urethritis, die nicht durch Neisserien (NGU) verursacht wird, und 3 % mit bakterieller Gastroenteritis entwickelt sich eine ReA [16, 34]. *Chlamydia trachomatis* ist dabei einer der häufigsten Ursachen [4]. Daten einer polnischen Arbeitsgruppe zeigten, dass bei bis zu 13 % der Patienten mit ReA eine *C.-trachomatis*-Infektion bestand [27]. Keat et al. untersuchten 531 Männer mit nichtspezifischer Urethritis. Bei 36 % der Patienten wurde *C. trachomatis* nachgewiesen. Davon entwickelten 3,6 % im Verlauf eine ReA, HLA-B27-positive Männer hatten dabei ein 10fach erhöhtes Risiko, eine SARA zu erleiden [22]. Ein Review von 2007 zeigte bei Patienten mit HLA-B27-Assoziation gar ein 50fach erhöhtes Risiko, an einer postinfektiösen ReA zu erkranken [1, 29].

Eine der wenigen Studien über sexuell übertragene Infektionserkrankungen bei Athleten stammt von de Araujo aus dem Jahr 2014. Es wurde die Prävalenz von sexuell übertragenen Erkrankungen (STD) bei Leistungssportlerinnen in Sao Paulo untersucht [2]: 24 von 50 Athletinnen wurden dabei positiv getestet. Das humane Papillomavirus wurde bei 44 % der Sportlerinnen und damit am häufigsten nachgewiesen. Zwei Athletinnen waren mit *C. trachomatis* infiziert. Infektionen mit Neisseria gonorrhoeae, Syphilis, Hepatitis B oder C sowie dem HI-Virus wurden nicht nachgewiesen.

Im Jahr 1922 beschrieb Patton eine Fallserie von acht professionellen Wrestlern mit Trachom nach Chlamydieninfektion. Zentmayer veröffentliche 1934 einen weiteren Fall [43]. Turbville identifizierte in einem Review 59 Infektionserkrankungen bei Leistungssportlern von 1922 bis 2005. Am häufigsten wurde Herpes simplex mit 22 % nachgewiesen. Sportarten mit der höchsten Inzidenz waren Wrestling, Rugby und Fußball [38].

## Anamnese und klinische Untersuchung

Zur Diagnosestellung der SARA ist eine sorgfältige Anamnese geboten. Ein Trauma wird in der Regel verneint, gelegentlich werden Bagatelltraumata beschrieben. Die Sexualanamnese ist essenziell. Schambedingte Falschangaben verzögern nicht selten die Diagnosestellung (vgl. Fall 2). Hinweise auf Promiskuität oder wechselnde Partnerschaften machen die Diagnose einer SARA wahrscheinlicher. Infektstigmata, Infektionserkrankungen und B‑Symptomatik müssen erfragt werden. Dennoch ist die Diagnosefindung nicht profan, da klinische Symptome von *C.-trachomatis*-Infektionen bei bis zu 70 % der weiblichen und 25 % der männlichen Patienten fehlen [45]. Die Diagnosestellung der SARA wird zudem nicht selten durch einen fehlenden Erregernachweis erschwert.

Es können Symptome von leichter Monarthralgie bis zum Maximalbild der chronischen Polyarthritis bestehen. Führend betroffen ist die untere Extremität, insbesondere das Kniegelenk [7]. Der Gelenkerguss ist in der klinischen Untersuchung gut nachvollziehbar und kann unmittelbar sonographisch bestätigt werden [30]. Lokale Infektstigmata sind zu prüfen (Differenzialdiagnose septische Arthritis). Der Befall des Achsskelettes, insbesondere der lumbalen Wirbelsäule, wie beim verwandten Morbus Bechterew ist selten [7, 13, 15]. Gleiches gilt für Symptome wie Achillodynie und Myalgien. Extraskelettale Manifestationen können als palmare bzw. plantare Psoriasis oder entzündlicher Prozesse der Augen (Uveitis, Iridozyklitis) auftreten [1, 13].

## Diagnostik

Der Erregernachweis bei Verdacht auf eine Chlamydieninfektion hat Priorität, da nur in diesem Fall eine antibiotische Therapie erfolgen sollte. Es bestehen verschiedene diagnostische Möglichkeiten zum Nachweis einer Chlamydieninfektion. Neben direkten Verfahren (mikrobiologische Kultur, molekularer DNA-Nachweis mittels PCR) stehen auch indirekte Nachweismöglichkeiten wie die serologische Antikörperbestimmung gegen *C. trachomatis* zur Verfügung.

Für den Erregernachweis in der Zellkultur sind genitale oder okuläre Abstriche (Urethra, Zervix, Anus, Konjunktiven) das Material der Wahl. Auch der kulturelle Nachweis im Urin ist grundsätzlich möglich, kann bei negativem Befund eine Infektion jedoch nicht ausschließen [21]. Auch wenn der Kulturnachweis eine hohe Spezifität aufweist, so gelingt ein Erregernachweis nur in 60–80 % der Fälle. Die Kultur wurde daher mittlerweile durch sensitivere molekulare Nachweisverfahren als diagnostischer Goldstandard abgelöst und gilt als veraltet. Als vorrangiges Diagnostikum sind aufgrund der höchsten Sensitivität von 97 % [39] mit vergleichbarer Spezifität von 99,4 % zur Kultur daher Nukleinsäureamplifikationstests wie die Polymerasekettenreaktion (PCR) zu wählen. Urethralabstrich und Urindiagnostik scheinen gleichwertig. Es sollte jedoch darauf geachtet werden, etwa 10–20 ml des Erststrahlurins abzufangen (nicht zwingend Morgenurin) [26, 41]. Als weiteres Untersuchungsmaterial für die PCR eignen sich durch Gelenkpunktion gewonnene Synovialflüssigkeit oder arthroskopisch gewonnene Gelenkschleimhaut. Taylor-Robinson et al. erbrachten bereits 1991 den Nachweis von Chlamydien-DNA in der Synovialmembran mittels PCR [36]. Da der direkte Chlamydiennachweis auch bei florider Infektion und o. g. Sensitivitätsraten misslingen kann, schließt ein fehlender Erregernachweis eine SARA dennoch nicht vollständig aus [42].

Bei akuter urogenitaler Infektion ist die Bestimmung serologischer Parameter aufgrund der zeitlichen Latenz bis zur Bildung von Antikörpern nach Antigenkontakt von bis zu 8 Wochen nicht sinnvoll. Dagegen ist die Bestimmung dieser Parameter (insbesondere Antikörpertiter wie IgA, IgG und IgM) zur Abklärung postinfektiöser Komplikationen, wie der reaktiven Arthritis, eine sinnvolle ergänzende Untersuchung [23]. Die serologische Determinierung von IgA, IgM und IgG gibt Hinweise auf eine akute bzw. persistente (IgM) oder bereits längerfristig stattgehabte Infektion mit *C. trachomatis* (IgG). Ein positiver Nachweis von IgM-Antikörpern macht eine floride Infektion sehr wahrscheinlich [23].

Unter prognostischem Aspekt bestehen Hinweise, dass eine überschießende Bildung von Antikörpern der Immunglobulinklasse G mit einem erhöhten Risiko für die Entwicklung einer SARA assoziiert ist [23]. Es sollten im untersuchenden Labor zudem Informationen eingeholt werden, ob die angebotenen Testverfahren zwischen den verschiedenen Chlamydienarten differenzieren können. Auch bei erfolgreich therapierter Urogenitalinfektion können hohe IgG-Antikörper langfristig nachweisbar bleiben. Insbesondere zur Therapiekontrolle nach antibiotischer Behandlung der urogenitalen Infektion oder der Aktivitätsbestimmung der ReA eigenen sich serologische Titerbestimmungen daher nicht [11]. Bas et al. konnten zeigen, dass IgA-Antikörper bei Chlamydien-induzierter SARA auch intraartikulär vorlagen [3]. Eine Indikation zur standardmäßigen Immunglobulinbestimmung aus der Synovialflüssigkeit kann hieraus dennoch nicht abgeleitet werden.

Eine ergänzende laborchemische Untersuchung von Infektparametern (Leukozyten, CRP, BSG) sollte durchgeführt werden, um Hinweise auf ein potenziell alternatives septisches Geschehen zu erhalten. Insbesondere bei der aseptischen ReA sind diese Parameter nicht selten normwertig und lassen durchaus eine Abgrenzung zu einem Gelenkempyem oder einer floriden rheumatoiden Arthritis zu [15].

Zur Komplettierung der Diagnostik ist insbesondere bei Leistungssportlern eine frühzeitige MRT-Bildgebung des betroffenen Gelenkes ratsam. Auf diese Weise können weitere Differenzialdiagnosen bei nicht erbrachtem Erregernachweis ausgeschlossen werden (z. B. die pigmentierte villonoduläre Synovialitis als proliferative Erkrankung der Synovialmembran, eine Gelenkchondromatose, freie Gelenkkörper oder anderweitige operativ zu adressierende Binnenschäden).

## Therapie der Primärinfektion

Die antibiotische Behandlung einer Chlamydien-induzierten Urogenitalinfektion ist sowohl bei symptomatischen als auch bei asymptomatischen Patienten obligat [4]. Die orale Therapie erfolgt mit dem bakteriostatisch wirksamen Doxycyclin (Klasse der Tetrazykline). Es weist eine gute Wirksamkeit gegen intrazelluläre Erreger auf [24]. Als leitliniengerechte Therapie wird bei asymptomatischer Infektion oder unkomplizierter Urethritis bei beiden Geschlechtern zweimal täglich die Einnahme von 100 mg Doxycyclin für 7 Tage empfohlen [26]. Doxycyclin zeichnet sich grundsätzlich durch eine gute Verträglichkeit aus. Bei Kontraindikationen kann jedoch auf die orale Einmalgabe von 1,5 g Azithromycin ausgewichen werden. Bei Prostatitis und Epididymitis kann alternativ die 14-tägige Gabe von Fluorchinolonen erfolgen [26]. Darüber hinausgehende dezidierte Empfehlungen zur antibiotischen Therapie können in der S2k-Leitlinie der AWMF nachgelesen werden (Stand 08/2016). Eine entsprechende Diagnosestellung und Behandlung sollten jedoch in der Regel nicht ohne urologische Beurteilung erfolgen. Die simultane Behandlung des Sexualpartners ist erforderlich, um rezidivierende gegenseitige Ansteckungen zu vermeiden („Ping-Pong-Effekt“). Daher sind Sexualpartner der letzten 6 Monate in das klinische Management mit einzubeziehen [26]. Patienten und Lebenspartner/innen müssen dringend darüber aufgeklärt werden, dass Chlamydieninfektionen eine potenzielle Ursache für Sterilität sind [18]. Die Prävention sekundärer Komplikationen, wie beispielsweisen eines unerfüllten Kinderwunsches, ist hierbei ein wesentlicher Aspekt [18, 26].

## Therapie der reaktiven Arthritis

Swierkot wies bereits 2003 auf den fehlenden diagnostischen und therapeutischen Goldstandard der SARA hin [35]. Der diagnostische Pfad ist durch die gute Nachweismöglichkeit von Chlamydien-DNA in der PCR der Synovialflüssigkeit mittlerweile jedoch standardisiert. Zudem haben sich einige therapeutische Maßnahmen etabliert. Zur symptomatischen Behandlung eignen sich (nichtsteroidale) Antirheumatika (z. B. Etoricoxib, Diclofenac, Ibuprofen) [17]. Physikalische Maßnahmen und Physiotherapie können ergänzt werden [25]. Bei therapierefraktären Verläufen ist alternativ eine orale Kortisonstoßtherapie oder eine lokale Instillation (z. B. von 40 mg Triamcinolon) sinnvoll [31]. Der vorherige Ausschluss einer bakteriellen Arthritis ist in diesem Falle jedoch unabdingbar. Vor der Kortisoninstillation ist zudem die entlastende Punktion sinnvoll. Das Punktat ist bei Infektverdacht oder Verdacht auf eine SARA laborchemisch (Zellzahl), mikrobiologisch (Zellkultur) und molekulargenetisch in der eubakterielle PCR zu untersuchen. Bei rezidivierendem bzw. persistierendem Gelenkerguss trotz Anwendung adäquater Therapiemaßnahmen sollte aufgrund der erhöhten Verletzungsgefahr eine strikte Karenz von High-Impact-Sportarten eingehalten werden, bis eine vollständige Beschwerdefreiheit besteht [42].

Eine chronifizierte reaktive Arthritis kann analog zu einer rheumatischen (Poly‑)Arthritis behandelt werden [29, 42]. Dies beinhaltet bei therapierefraktären Verläufen eine Therapie mit den gleichen immunmodulierenden Medikamenten (DMARD) [40]. Zur Anwendung können in diesen Fällen in enger rheumatologischer Rücksprache beispielsweise Substanzen wie Methotrexat, Leflunomid oder Infliximab als TNF-alpha-Blocker kommen. Die Behandlung sollte daher unter fachärztlicher rheumatologischer Betreuung erfolgen. Wechalekar et al. veröffentlichten 2010 einen Fallbericht über die Behandlung einer therapierefraktären reaktiven Arthritis mit dreimaliger Infusion von Infliximab [40]. Die betroffene Patientin war anschließend dauerhaft beschwerdefrei und konnte eine bis dato verabreichte Basistherapie absetzen. Erfahrungen mit dieser Substanzklasse im Leistungssport liegen nicht vor. Die Anwendung außerhalb von Studien ist jedoch nicht zu empfehlen.

Sieper et al. untersuchten zur kurativen Behandlung der reaktiven Arthritis die Wirkung einer 3‑monatigen antibiotischen Therapie mit Ciprofloxacin gegenüber einer Placebobehandlung [33]. Ein signifikanter Nutzen dieser Behandlung wurde nicht nachgewiesen. Es bestand die Tendenz, dass, anders als nach gastrointestinalen Infekten, insbesondere Patienten mit einer Chlamydien-induzierten SARA von dieser Behandlung profitierten. Eine generelle Anwendung dieser Therapie kann aktuell jedoch bei fehlender Evidenz außerhalb von Studien nicht empfohlen werden. Es besteht bei dieser langfristigen antibiotischen Therapie unter anderem das Risiko einer Selektion hochresistenter Keime aus der Normalflora des Patienten und einer Störung der Zusammensetzung des Mikrobioms bis hin zu einer pseudomembranösen Kolitis. Carter et al. erhielten 2010 in einer Doppelblindstudie Hinweise darauf, dass die Einnahme einer Antibiotikakombination aus Doxycyclin, Rifampicin und Azithromycin über 6 Monate eine effektive Therapie für die chronische SARA bedeuten kann [6]. Auch hier ist jedoch von einer generellen Anwendung dieser Therapie bei bisher fehlendem Nachweis der Evidenz abzuraten. Aufgrund der geringen Stoffwechselaktivität der Elementarkörperchen und der langsamen Vermehrung der aberrierenden Retikularkörperchen sind Chlamydien nur sehr eingeschränkt durch eine konventionelle antibiotische Therapie adressier- und eradizierbar. Diese Eigenart der Chlamydien begünstigen allerdings das nicht seltene Phänomen der lokalen Infektpersistenz der reaktiven Arthritis [6, 42].

Im Falle einer Augenbeteiligung mit Regenbogenhautentzündung (Iritis), hat zwingend eine ophthalmologische Mitbehandlung zu erfolgen. Nur eine frühzeitige Therapie kann dann gravierende Folgeschäden, wie dauerhafte Einschränkungen der Sehfähigkeit, verhindern [13].

Prognostisch relevant – insbesondere auch für den ambitionierten Sportler – ist die Erkenntnis, dass eine SARA trotz multimodaler Therapie bis zu 6 Monate und länger klinisch symptomatisch sein kann [7, 42]. Einem Fünftel der Betroffenen droht sogar eine langfristige Chronifizierung [15]. Sollte sich das Erkrankungsbild trotz der o. g. Behandlung therapierefraktär zeigen, stellt die arthroskopische Synovialektomie eine invasive, aber erfolgversprechende therapeutische Option dar [4, 28].

## Diskussion

Die Infektion mit *C. trachomatis* und nachfolgender postinfektiöser reaktiver Arthritis kann trotz frühzeitiger Diagnosestellung und adäquater Therapie aufgrund potenziell langer Ausfallzeiten eine einschneidende Erkrankung in einer Sportlerkarriere sein. Während die primäre Urogenitalinfektion antibiotisch gut therapierbar ist, fehlt ein therapeutischer Goldstandard derzeit nicht nur für Leistungssportler. Daher wird die Behandlung der Synovialitis weitestgehend individuell durchgeführt wird. Langfristiges Ziel sollte es daher sein, einen standardisierten Therapiepfad zu definieren. Die Berücksichtigung der aktuellen S2k-Leitlinie der AWMF (*Infektionen mit Chlamydia trachomatis*, Stand 2016) kann hierzu einen wesentlichen Beitrag leisten.

Bemerkenswert ist die aktuell weiterhin spärliche Datenlage zur reaktiven Arthritis nach Chlamydieninfektion, insbesondere im Leistungssport. Daher überrascht es nicht, dass in der Literatur für diese Patientengruppe keine klaren Therapieempfehlungen oder Daten über Ausfallzeiten im Sport vorhanden sind. Eigene Erfahrungen zeigen durchaus hartnäckige klinische Verläufe. Der geschilderte zweite Fall zeigt, dass ohne aufrichtige Angaben in der Anamnese die weitere Diagnostik und Therapie sehr protrahiert verlaufen kann, da die adäquate Diagnostik in diesen Fällen nicht initiiert wird. Mit der entsprechenden Anamneseerhebung und etwas klinischer Erfahrung ist sowohl die urogenitale Primärinfektion als auch die SARA jedoch gut und zielgerichtet zu diagnostizieren und zu therapieren. Bei Vorliegen eines atraumatischen Gelenkergusses ohne anderweitig erkennbare Ursache sollte daher im jungen Patientenkollektiv frühzeitig zu einer Eukaryonten-PCR des Gelenkpunktates tendiert werden.

Therapierefraktäre Verläufe mit Persistenz der reaktiven Arthritis sind allerdings aufgrund des speziellen Replikationsmechanismus der Chlamydien und der Fähigkeit, sich teilweise der Immunantwort des Wirtes entziehen zu können, keine Seltenheit. Hierbei ist noch ungeklärt, inwiefern hierdurch chronische Gelenkschäden oder organische Komplikationen hervorgerufen werden können.

Bei rezidivierenden Arthritiden ist an eine potenziell asymptomatisch verlaufende urogenitale Infektpersistenz oder eine Reinfektion zu denken. Dann sind die erneute urologische Diagnostik und entsprechende Behandlung einzuleiten.

## Fazit für die Praxis

- Die postinfektiöse reaktive Arthritis nach urogenitaler Infektion mit *C. trachomatis* ist eine wichtige Differenzialdiagnose bei atraumatischer Gelenkschwellung.
- Die Erhebung der Sexualanamnese ist ein wesentlicher Aspekt zur zielgerichteten Diagnosestellung der SARA (sexuell übertragene postinfektiöse Arthritis) und ist insbesondere im jungen Patientenkollektiv mit wechselnden Partnerschaften unverzichtbar.
- Der direkte Nachweis von *C. trachomatis* ist bei urogenitaler Infektion im Erststrahlurin oder im Urethralabstrich zuverlässig mittels PCR (Polymerasekettenreaktion) nachweisbar.
- Die Erstlinientherapie erfolgt oral mit 100 mg Doxycyclin zweimal täglich für 7 Tage und schließt die Mitbehandlung des Sexualpartners ein.
- Goldstandard zur Diagnose der postinfektiösen reaktiven Arthritis ist der direkte Erregernachweis aus dem Gelenkpunktat in der PCR.
- Neben einer symptomatisch-antiphlogistischen Behandlung der Gelenkbeteiligung besteht bei therapierefraktären Verläufen die Möglichkeit der arthroskopischen partiellen Synovialektomie, sowie die Verwendung von Basistherapeutika unter rheumatologischer Betreuung
- Für eine antibiotische Therapie der akuten oder chronischen reaktiven Arthritis bei fehlendem urogenitalem Keimnachweis besteht derzeit keine Evidenz.
- Die Bestimmung von Antikörpertitern gehört nicht zur Standarddiagnostik der SARA und eignet sich allenfalls zum Nachweis eines Erregerkontaktes mit *C. trachomatis* und anderweitig fehlendem Erregernachweis. Für Therapiekontrollen eignen sie sich jedoch nicht.

## Abkürzungen

AWMF: Arbeitsgemeinschaft der Wissenschaftlichen Medizinischen Fachgesellschaften
BSG: Blutsenkungsgeschwindigkeit
CRP: C‑reaktives Protein
DMARD: Disease modifying anti-rheumatic drugs (Basistherapeutika in der Rheumatologie)
HLA-B27: Humanes Leukozytenantigen B27
Ig: Immunglobulin
NGU: Nicht-Gonokokken-Urethritis
NSAR: Nichtsteroidales Antirheumatikum
PCR: Polymerasekettenreaktion
ReA: Reaktive Arthritis
SARA: Sexuell übertragene postinfektiöse Arthritis
STIR: Short tau inversion recovery (Spezielle MRT-Sequenz)
TNF: Tumornekrose-Faktor
